# Supplementary material for: Development of the Active Ageing Awareness Questionnaire in Malaysia
Source: Healthcare (Basel). 2021 Apr 22;9(5):499. doi: 10.3390/healthcare9050499 (PMC8146047; doi:10.3390/healthcare9050499)
Supplement: Supplementary file 1 [file healthcare-09-00499-s001.zip › healthcare-1109158-supplementary.pdf]

healthcare

MDPI

Article

Development of the Active Ageing Awareness Questionnaire in Malaysia

Nor Hana Ahmad Bahuri [1], Hussein Mohammad Rizal [2], Hazreen Abdul Majid [2], Mas Ayu Said[3]* and Tin Tin Su [4]

1 Department of Social and Preventive Medicine, Faculty of Medicine, University of Malaya, 50603 Kuala Lumpur, Malaysia; drhannadrph@gmail.com (N.H.A.B)
2 Centre for Population Health (CePH), Department of Social and Preventive Medicine, Faculty of Medicine, University of Malaya, 50603 Kuala Lumpur, Malaysia; husseinriz@um.edu.my (H.R.); hazreen@ummc.edu.my (H.A.M.)
3 Centre for Epidemiology and Evidence-Based Practice, Department of Social and Preventive Medicine, Faculty of Medicine, University of Malaya, 50603 Kuala Lumpur, Malaysia
4 South East Asia Community Observatory (SEACO), Jeffrey Cheah School of Medicine & Health Sciences, Monash University, Bandar Sunway, 47500 Subang Jaya, Selangor, Malaysia; TinTin.Su@monash.edu (T.T.S)
* Correspondence: mas@ummc.edu.my

Supplementary Table S1. English and Malay translation and reasons for exclusion of the Awareness of Active Ageing Questionnaire (AAAQ)

Item No.	Item Text (English)	Item Text (Malay)	I-CVI[a]	ICC[b]	EFA[c]	CFA[d]	Reason for Exclusion
**1**	**In your opinion, what are the factors that make someone age actively?**	**Pada pendapat anda, apakah faktor yang membolehkan seseorang itu menua secara aktif?**	**1.00**	**-**	**-**	**-**	**Included (open-ended item)**
2	In Malaysia, at what age a person is called as an 'older person'?	Di Malaysia, pada umur berapakah seseorang itu dikatakan 'warga emas'?	0.67	-	-	-	Excluded I-CVI consensus (open-ended item)
**3**	**Have you ever heard about 'active ageing'?**	**Pernahkah anda mendengar mengenai 'penuaan aktif'?**	**1.00**	**-**	**-**	**-**	**Included (open-ended item)**
**4**	**Free from chronic diseases such as diabetes mellitus or heart disease**	**Bebas dari penyakit kronik seperti kencing manis atau penyakit jantung**	**1.00**	**0.634**	**0.586**	**0.55**	**Included**
5	Chronic diseases or diseases which is/are under controlled	Mempunyai penyakit kronik yang terkawal	1.00	0.628	0.239	-	Low factor loading for CFA
**6**	**Able to move around freely with or without disability or physical limitation**	**Tidak mempunyai masalah untuk bergerak ke mana-mana tidak kira samada mempunyai kecacatan anggota / masalah fizikal atau pun tidak**	**1.00**	**0.696**	**0.601**	**0.59**	**Included**
**7**	**Regular physical activity or exercise**	**Melakukan aktiviti fizikal atau senaman secara berkala**	**1.00**	**0.486**	**0.779**	**0.77**	**Included**
8	Not smoking (cigarette, e-cigarette, pipes, cigar)	Tidak merokok (rokok dan "vaping")	1.00	0.788	0.654	-	Excluded, modification indices more than 15 indicate redundant with covariates
**9**	**Eating healthy food**	**Makan makanan yang sihat**	**1.00**	**0.624**	**0.882**	**0.87**	**Included**
**10**	**Not consuming alcoholic beverages**	**Tidak mengambil minuman beralkohol**	**1.00**	**0.722**	**0.821**	**0.84**	**Included**
**11**	**Good oral health**	**Tiada masalah kesihatan gigi**	**1.00**	**0.712**	**0.738**	**0.75**	**Included**
**12**	**Healthy minds**	**Mempunyai minda yang sihat**	**1.00**	**0.690**	**0.886**	**0.86**	**Included**
13	Good memory	Daya ingatan yang bagus	1.00	0.584	0.867	-	Excluded cross-loading EFA
**14**	**Not feeling lonely**	**Tidak merasa kesepian**	**1.00**	**0.677**	**0.754**	**0.74**	**Included**
15	Continue working (part-time or full time) after compulsory retirement	Meneruskan pekerjaan selepas tempoh persaraan wajib	1.00	0.751	0.340	-	Low factor loading for CFA

**16**	**Participate in voluntary activity. For example, volunteer in Flood Relief Team**	**Menyertai aktiviti kemanusiaan. Contohnya sukarelawan Bantuan Mangsa Banjir**	**1.00**	**0.629**	**0.498**	**0.63**	**Included**
**17**	**Participate in social and community activity**	**Terlibat dengan aktiviti sosial dan kemasyarakatan**	**1.00**	**0.737**	**0.687**	**0.76**	**Included**
18	Providing care for other older person	Menjaga orang yang lebih tua dari kita	1.00	0.739	0.417	-	Low factor loading for CFA
19	Providing care for grandchildren	Menjaga cucu	1.00	0.754	0.064	-	Low factor loading for CFA
**20**	**Having a hobby such as gardening, fishing or baking**	**Mempunyai hobi seperti berkebun, memancing dan membuat kuih**	**1.00**	**0.728**	**0.742**	**0.82**	**Included**
**21**	**Continue learning new things**	**Sentiasa belajar perkara-perkara baru**	**1.00**	**0.608**	**0.742**	**0.81**	**Included**
**22**	**Have a good amount of saving for retirement**	**Mempunyai wang simpanan yang mencukupi untuk bersara**	**1.00**	**0.540**	**0.825**	**0.73**	**Included**
**23**	**Have own house**	**Mempunyai tempat tinggal sendiri**	**1.00**	**0.787**	**0.923**	**0.82**	**Included**
24	Safe living conditions	Tempat tinggal yang selamat	1.00	0.691	0.974	-	Excluded, modification indices more than 15 indicate redundant with covariates
25	Safe neighbourhood	Persekitaran kejiranan yang selamat	1.00	0.699	0.956	-	Excluded cross-loading EFA

Item Content Validity Index (I-CVI)—number of experts rating of three total number of experts.

[a]— Items with I-CVI ≤ 0.69 were excluded, 0.69 < I-CVI < 0.90 were individually assessed, I-CVI ≥ 0.90 were retained

Inter-item Correlation (ICC)—test-retest were conducted to assess the initial reliability of the questionnaire

[b]— Items with ICC was classified as: poor (< 0.4), fair to good (0.4 to < 0.75) and excellent (≥ 0.75)

Exploratory Factor Analysis

[c]—Bartlett's test of sphericity with a significance level of less than 0.5 and a KMO of more than 0.7 or nearly 1.0 indicate that the items are suitable for structure detection and factor analysis and are useful for further analysis

Confirmatory Factor Analysis

[d]— Dimensionality is achieved when all the items have a factor loading of more than 0.5

Bold items are included in the final AAAQ. The grey cells indicate that items were excluded in the validation process.

Item 4-25— Items are rated according to a 4-point Likert scale from strongly disagree to strongly agree on what makes someone age actively
